# Automatic Extraction of Mental Health Disorders From Domestic Violence Police Narratives: Text Mining Study

**DOI:** 10.2196/11548

**Published:** 2018-09-13

**Authors:** George Karystianis, Armita Adily, Peter Schofield, Lee Knight, Clara Galdon, David Greenberg, Louisa Jorm, Goran Nenadic, Tony Butler

**Affiliations:** 1 Kirby Institute Faculty of Medicine University of New South Wales Sydney Australia; 2 Neuropsychiatry Service Hunter New England Health Newcastle Australia; 3 Victims Services New South Wales Department of Justice Sydney Australia; 4 School of Psychiatry University of New South Wales Sydney Australia; 5 Centre for Big Data Research in Health University of New South Wales Sydney Australia; 6 School of Computer Science University of Manchester Manchester United Kingdom

**Keywords:** text mining, rule-based approach, police narratives, mental health disorders, domestic violence

## Abstract

**Background:**

Vast numbers of domestic violence (DV) incidents are attended by the New South Wales Police Force each year in New South Wales and recorded as both structured quantitative data and unstructured free text in the WebCOPS (Web-based interface for the Computerised Operational Policing System) database regarding the details of the incident, the victim, and person of interest (POI). Although the structured data are used for reporting purposes, the free text remains untapped for DV reporting and surveillance purposes.

**Objective:**

In this paper, we explore whether text mining can automatically identify mental health disorders from this unstructured text.

**Methods:**

We used a training set of 200 DV recorded events to design a knowledge-driven approach based on lexical patterns in text suggesting mental health disorders for POIs and victims.

**Results:**

The precision returned from an evaluation set of 100 DV events was 97.5% and 87.1% for mental health disorders related to POIs and victims, respectively. After applying our approach to a large-scale corpus of almost a half million DV events, we identified 77,995 events (15.83%) that mentioned mental health disorders, with 76.96% (60,032/77,995) of those linked to POIs versus 16.47% (12,852/77,995) for the victims and 6.55% (5111/77,995) for both. Depression was the most common mental health disorder mentioned in both victims (22.25%, 3269) and POIs (18.70%, 8944), followed by alcohol abuse for POIs (12.19%, 5829) and various anxiety disorders (eg, panic disorder, generalized anxiety disorder) for victims (11.66%, 1714).

**Conclusions:**

The results suggest that text mining can automatically extract targeted information from police-recorded DV events to support further public health research into the nexus between mental health disorders and DV.

## Introduction

Domestic violence (DV) can be defined as “any incident of threatening behavior, violence, or (psychological, physical, sexual, financial, emotional) abuse between adults who are or have been an intimate partner or family member, regardless of gender or sexuality” [1]. DV can also occur in other relationships, such as between a caregiver and a dependent person or those living together in a household in a nonintimate relationship [2]. It is recognized as one of the most common forms of interpersonal violence and is an international social and public health problem with important health care consequences affecting the lives of thousands, mostly women, each year [3-5]. According to the World Health Organization’s (WHO) multicountry study of violence, the prevalence of physical and sexual partner violence toward women ranges from 15% to 71% globally [3,5]. In 2014, almost 50,000 people in Australia were recorded by the police as the victims of DV [6]. The cost of DV is significant with estimates suggesting that, in Australia, the cost of violence against women was approximately Aus $22.2 billion in 2015-2016, and in the United Kingdom and the United States, £17 billion and US $4.1 billion, respectively [4,5,7].

Domestic violence shares a complicated relationship with the onset, duration, and recurrence of mental health disorders, including substance abuse, eating disorders, posttraumatic stress, and suicidal tendencies, as well as exacerbation of psychotic symptoms [3-5,8]. Previous reports have suggested an increased risk of DV in populations with mental health disorders in comparison to those with no mental illness [3,9]. Over the past 20 years, a consensus has emerged that there is a modest (yet statistically significant) relationship between severe mental illness and violence, with severe mental illness increasing the risk of an individual to be violent toward others [10].

In 2017, the New South Wales Police Force (NSWPF) recorded 123,330 DV-related events in WebCOPS, a Web-based interface for the Computerised Operational Policing System (COPS) that enables the police to capture and analyze crime information on an organization-wide basis, with approximately 37% resulting in an offense being recorded (NSW Police Force, personal communication, June 2018). Information about DV events contained in WebCOPS is available as both structured form (eg, fields documenting information such as date of birth, Aboriginal status, whether weapons were used) and free unstructured text (“event narratives”). Each event contains one or more text narratives that describe in detail the alleged incident(s) that occurred between the person of interest (POI) and the victim, the circumstances of the event, and any action(s) taken by the police. The narratives are often written without a specific structure, populated with frequent misspellings and typographical errors, often with (sometimes informal) acronyms and abbreviations that can bear ambiguous meaning depending on the context.

The large number of DV events and the associated text narratives, however, prevent the extraction of potentially useful information using traditional ethnographic/qualitative approaches. One recent research paper commented that “...there is no systematic way to extract information from these [police] narratives other than by manual review” [11].

Still, automated methods for large-scale processing of free text known as *text mining* have been used for over 30 years to harvest information from unstructured text in many domains, particularly in biomedicine [12-15]. Recent attempts have aimed to utilize text mining to identify crime-related information from online media publications [16,17]. However, few efforts have been conducted in processing police reports [18-20]. Limited work included identification of offenders’ names, narcotic drugs, and weapons with various degrees of success (F-score [a measure of a method’s accuracy] ranging from 46% to 81%) through named entity extractors [18,19] and classification of police reports as DV or non-DV related by applying an unsupervised clustering technique that classified 44% of the reports set aside for manual inspection correctly [20].

Several attempts have been also made to extract mental health-related information from various free-text resources [21-27]. For example, drug side effects were extracted from psychiatric narratives by applying either hybrid methodologies of machine learning and dictionaries with rules, or rule-based approaches only that returned F-scores between 75% and 85% [21,24,25]. Treatment outcomes for major depressive disorders were identified from electronic medical records using a supervised approach with logistic regression, with precision ranging from 78% to 86% [22]. Mini Mental State Examination results were recognized from clinical notes and health record correspondence between clinicians with 85% and 91% F-scores, respectively, through a rule-based method [23]. Jackson et al [26] and Karystianis et al [27] both identified symptoms of mental illness from clinical discharge summaries and psychiatric records using either regular expression pattern matching or a rule-based approach with 88% and 81% F-scores, respectively [26,27].

In this paper, we examine whether automatic text mining of DV police event narratives is feasible in identifying mentions of mental health disorders at the narrative level among those involved in DV events by employing a knowledge-driven approach. This approach is based on lexicalized rules combined with manually constructed dictionaries that characterize mental health disorders in both POIs and victims involved in domestic disputes recorded by the NSWPF. We further perform a large-scale analysis of 492,393 DV events and report the results. To our knowledge, there has not been any application of text mining in the area of DV using real-world events and this is the first attempt of its kind to capture important mental health information in a large-scale analysis of DV events as recorded by the police.

## Methods

### Overview

Mentions of mental health disorders (including traumatic brain injury) were identified among POIs and victims in DV disputes based on the full list of the disorders (Textbox 1) according to the WHO’s *International Classification of Diseases, Tenth Revision* (*ICD-10*) for mental and behavioral disorders [28]. We also recognized mentions of unspecified mental disorders reported in the narratives (eg, “the defendant has mental health issues,” “victim is suffering from a severe mental disorder”), mentions of psychotropic medications by name or drug class (eg, “the victim takes Valium,” “accused takes a number of antidepressants”), and mentions of traumatic brain injury, drug prescription abuse, substance abuse, and drug-induced disorders.

### Data

We obtained records of 492,393 DV events from WebCOPS from January 2005 to December 2016 flagged either as “domestic violence related” or the description of violence in WebCOPS was coded as “domestic” or the relationship between the victim and the POI included any of the following: spouse/partner (including ex-spouse/ex-partner), boyfriend/girlfriend (including ex-boyfriend/ex-girlfriend), parent/guardian (including step/foster), child (including step/foster), sibling, other member of family (including kin), or carer. These events covered the following categories: various types of assaults, breaches of Apprehended Violence Orders, homicides, malicious damage to property, and offense against another person such as intimidation, kidnapping, abduction, and harassment. The records also contained incidents where no crime was committed but the police attended the DV event nonetheless. All event narratives contained personal information (eg, first name, surname, address) and therefore are not available to the general public. Permission to access the narratives was granted by the NSWPF following ethics approval from the University of New South Wales Human Research Ethics Committee (reference: HC16558) with access limited only to some authors of this study (GK, AA, TB). Strict security protocol ensured that text mining of the narratives could only be undertaken on site at the NSWPF headquarters and only deidentified extracted outputs could be taken off-site. A hypothetical example of a deidentified narrative is shown in Multimedia Appendix 1.

We randomly selected 100 events containing mental health disorder mentions for our training set, and an extra set of 100 other randomly chosen ones as a development set to optimize the performance of the text-mining system.

**Mental health disorders listed in the International Classification of Diseases, Tenth Revision (ICD-10) including the eight new categories targeted for extraction in domestic violence events and examples as they appeared in the police event narratives.**

1. Mental disorders due to known physiological conditions: eg, vascular dementia, unspecified dementia
2. Mental and behavioral disorders due to psychoactive substance abuse: eg, alcohol-related disorders, cannabis addiction, nicotine dependence
3. Schizophrenia, schizotypal, delusional, and other non-mood psychotic disorders: eg, schizophrenia, delusions, schizoaffective disorder
4. Mood (affective) disorders: eg, manic episodes, bipolar disorder, depression
5. Anxiety, dissociative, stress-related, somatoform, and other nonpsychotic mental disorders: eg, phobia, dissociative disorder, body dysmorphic disorder
6. Behavioral syndromes associated with physiological disturbances and physical factors: eg, eating disorders, bulimia, anorexia
7. Disorders of adult personality and behavior: eg, paranoid personality disorder, borderline personality disorder, kleptomania
8. Intellectual disabilities: eg, intellectual disability, severe intellectual disability
9. Pervasive and specific developmental disorders: eg, autism, mathematics disorder, phonological disorder
10. Behavioral and emotional disorders with onset usually occurring in childhood and adolescence: eg, attention deficit hyperactivity disorder, antisocial personality disorder, transient tic disorder
11. Unspecified mental disorder: eg, mental health issues, mental condition, mental health problem
12. Intentional self-harm: eg, self-harm, cut herself on purpose, self-harming issues
13. Injury of unspecified body region: eg, suicide attempt, multiple suicide attempt, attempted to kill himself
14. Symptoms, signs, and abnormal clinical and laboratory findings: eg, suicidal ideation, suicidal thoughts, suicidal tendencies
15. Other degenerate diseases of the nervous system: eg, Alzheimer disease, frontotemporal dementia
16. Chromosomal abnormalities not elsewhere classified: eg, Down syndrome
17. Drug prescription abuse: eg, addiction in prescribed medications, abusing prescribed medication
18. Traumatic brain injury: eg, brain damage, serious brain injury, brain trauma
19. Substance abuse: eg, substance abuse problem, ongoing drug abuse problems
20. Mental health medications antipsychotics: eg, Clozapine, antipsychotic medications, Risperdal
21. Mental health medications neuroleptics: eg, neuroleptic medications, neuroleptic drugs
22. Mental health medications antianxiety: eg, Xanax, medications: anxiety, Alprazolam
23. Mental health medications antidepressants: eg, Escitalopram, Anafranil, antidepressant medication
24. Unspecified drug-induced disorders: eg, drug-induced disorder, drug-induced mental health problem
25. Unspecified diseases of the nervous system: eg, neurological disorder
26. Systemic atrophies primarily affecting the central nervous system: eg, Huntington disease

#### Knowledge-Based System Development

**Figure 1:**
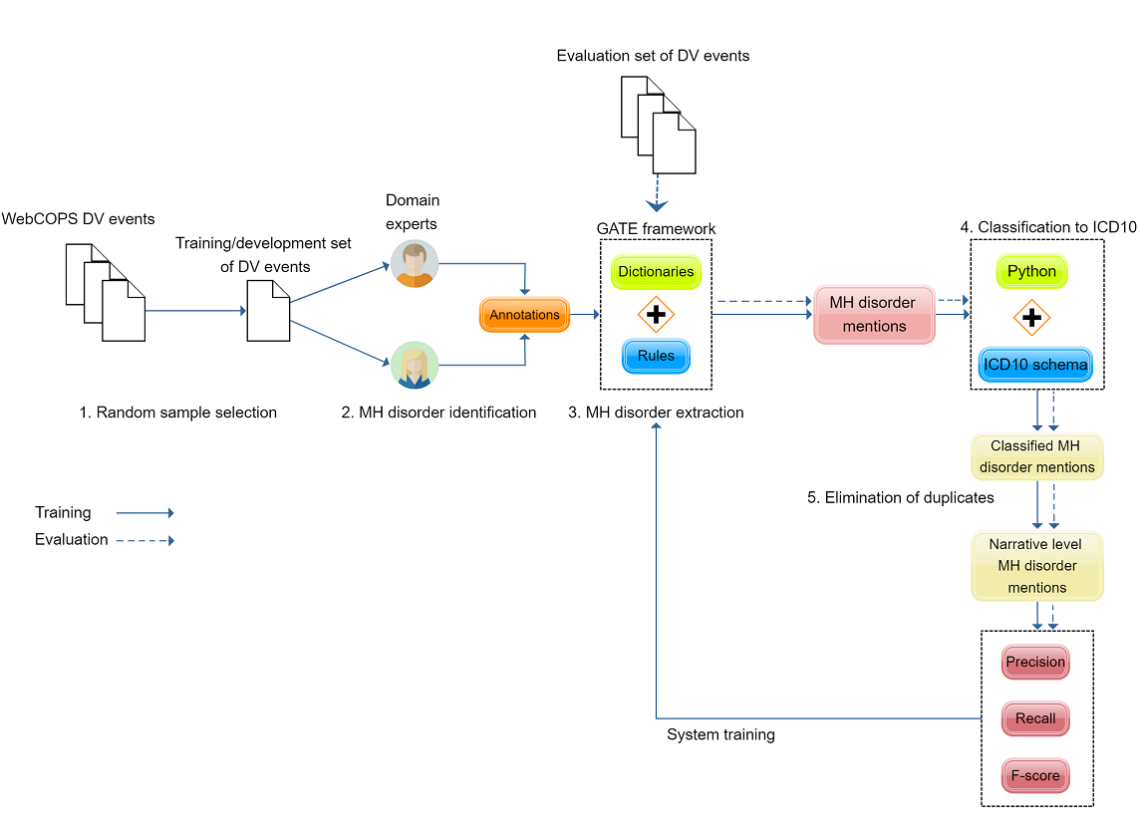
Overview of the text-mining approach used for the identification of mental health (MH) disorder mentions in domestic violence (DV)-related police event narratives. GATE is used as the environment for the rule design and application to mental health disorder mention identification. ICD-10: International Classification of Diseases, Tenth Revision.

Our approach involved the design and implementation of rule-based language expression patterns combined with term dictionaries to identify mentions of mental health disorders in both POIs and victims involved in DV events at the narrative level (see Figure 1 for an overview).

Our text-mining methodology consisted of the following steps (Figure 1):

1. Creation of specific dictionaries relevant to mental health disorders;
2. Design and implementation of rules to capture mental health disorder mentions in text;
3. Standardization and mapping of the extracted mental health disorder mentions into *ICD-10*; and
4. Elimination of duplicate mentions in each narrative to reach narrative level unification.

#### Dictionaries

Mentions of several task-specific semantic groups were identified through a set of custom-made dictionaries. The dictionaries were manually tailored by examining the training and development sets for the use of terms describing the associated mental health disorder mentions as well as expressions related to these conditions. For the identification of mental health disorders, we made use of terms and synonyms from the *ICD-10*, as well as common misspellings (eg, “schitzophrenia,” “aspergus syndrome”) or other indicative descriptive sentences (eg, “abuses alcohol,” “anger issues”) that were present in the event reports. A total of 13 dictionaries were crafted by the first author, GK (Table 1).

#### Rules

After inspecting the training set, we based our rules on lexical patterns in the text that indicated the presence of a mental health disorder for the POI, the victim, or both in a DV event. In the following example of a lexical pattern observed in a DV event (“accused is suffering from schizophrenia”) to identify a mental health disorder mention (“schizophrenia”), the word “accused” (the POI) is matched via a dictionary that contains variations of terms representing a POI (see Table 1) in which “is suffering from” is a semifrozen expression for the identification of the mental health disorder mention and “schizophrenia” gets a match through a dictionary containing various terms of mental health disorders (official and unofficial ones). The lexical patterns make use of (1) frozen lexical expressions as anchors for certain elements that are built through specific verbs, noun phrases, and prepositions (eg, “defendant suffers from”); and (2) semantic place holders (identifiable through the application of the manually crafted dictionaries (eg, all potential synonyms characterizing an individual as a victim such as “victim,” “vic,” “pn”) suggesting the presence of a mental health disorder. 

**Table 1:** Manually crafted dictionaries and their size (number of terms included) used to identify mental health disorder mentions.

Dictionary name	Size, n	Description	Examples
Adjectives	84	Adjectives indicating a mental disorder	alcoholic, schizophrenic, bipolar, autistic
Be	4	Conjugations of the verb “be” in present and past tense	is, was, were, are
Drug addiction	58	Illegal drugs known to cause addiction	cannabis, heroin, methamphetamines, ice
Drug names	228	Prescribed medications used to treat mental health disorders	Xanax, Valium, Stelazine, Tensium
Drug types	26	Medication classes used in treating mental health disorders	antianxiety, antidepressants, antipsychotic, mood stabilizer
Family	31	Terms indicating a family relationship	cousin, father, mother, grandfather
Have	5	Conjugations of the verb “have” in present and past tense	has been, have, having, has had
History	20	Variations of mental health history mentions	a short history of, hx, serious history of, extensive h/o
Mental disorder	594	Mental health disorder terms as appearing in the International Classification of Diseases, Tenth Revision (ICD-10) including traumatic brain injury and dementia, as well as unofficial terms, abbreviations, and synonyms observed in the police events	mood disorder, suicidal tendencies, split personality disorder
Negation	11	Terms indicating negated context	not, denies, none
Person of interest (POI)	18	Terms that describe a POI in a domestic violence event	defendant, POI, POI accused
Verbs	75	Verbs appearing in common lexical patterns that indicate a mental health disorder for POIs and victims	admitted, struggles, suffering, appears
Victim	19	Terms describing a victim in a domestic violence event	victim, vic, pn, pinop

Concept enumeration was also implemented because it appears frequently in the training data (eg, “POI has a history of depression, self-harm, and suicidal tendencies [mental health disorder mentions for POI]”). More than one lexical pattern may be matched in an event report and may refer to one or more disorder mentions (that can be duplicates) for the victim, the POI, or both.

**Figure 2:**
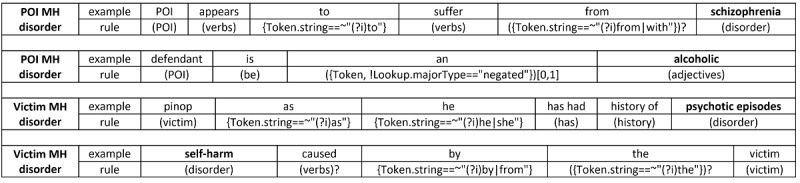
Rule examples (using GATE notation) for the recognition of mental health (MH) disorder mentions of persons of interest (POIs) and victims in domestic violence events. The identified disorder mentions are highlighted in bold.

For the generation and implementation of the rules, we used General Architecture for Text Engineering (GATE) [23], a text-mining framework for annotating and categorizing text that enables the identification of targeted information. GATE was chosen due to its support for rule-based text-mining approaches. The observed patterns in the text were converted into rules using the Java Annotations Pattern Engine (JAPE), a pattern matching language for GATE. A total of 264 rules were created with 137 for the POI and 127 for the victim, respectively. Figure 2 displays rule examples for the identification of mental health disorders.

The rules use lenient token matching (lowercase or uppercase), such as {Token.string==~”(?i)to”} matches “to”; various dictionaries contain variants, abbreviations, and synonyms of terms of interest, such as (victim), (POI), and (verbs) contain terms for victims, POIs, and verbs in various forms and tenses that describe victims or POIs suffering from a mental health disorder, respectively (see Table 2); ({Token!Lookup.majorType==”negated”})[0,1] will match any token that is not a part of the dictionary “Negated” (which contains negated indicators such as “not”); and the presence of “?” at the end of a rule component suggests its nonconditional nature (ie, it can appear or not in the text).

#### Mapping of Extracted Mental Health Disorder Mentions to the International Classification of Diseases, Tenth Revision

Since the extracted mental health disorder mentions are highly variable (synonyms, misspellings), any further analysis requires them to be mapped into standard mental health concepts such as the *ICD-10* mental and behavioral disorder categories. This was done automatically through a heuristic algorithm that relies on groups of terms that are representative of various *ICD-10* categories. If a given mention matched one term from a specific *ICD-10* category, then it was mapped to that category.

The mapping was done at four levels (see Multimedia Appendix 2). The first level was the most generic (26 categories), representing the overall type of mental health disorders as specified by *ICD-10* (see Textbox 1). The original *ICD-10* was expanded using eight customized categories to map mentions for which no direct mapping was obvious. Four of these eight categories involved mentions of psychotropic medications (“medications-antidepressants,” “medications-antianxiety,” “medications-antipsychotics,” “medications-neuroleptics”). For example, in event narratives where a medication class (eg, antidepressant medication) or a brand name (eg, “Zoloft”) was specified, we mapped them to a category called “medications-antidepressants.” The other four categories included “drug prescription abuse,” “substance abuse (unspecified),” “traumatic brain injury,” and “unspecified drug-induced disorder.” Cases in which we recognized that either the victim or the POI had an unknown mental health disorder or an unknown drug-induced mental disorder, were assigned into the categories of “unspecified mental disorder” or “unspecified drug-induced disorder,” respectively.

**Table 2:** Examples of extracted mental health disorder mentions (including misspellings) mapped into the International Classification of Diseases, Tenth Revision (ICD-10) schema. Note the inclusion of extra defined categories, such as “medications-antidepressants.”

Extracted mental health disorder mention	Standardized mental health disorder	ICD-10		
		First level	Second level	Third level
Oppositional defiant disorder	Oppositional defiance disorder	Behavioral and emotional disorders with onset usually occurring in childhood and adolescence	Conduct disorders	Oppositional defiance disorder
Intellectual disability	Intellectual disability	Intellectual disabilities	Intellectual disability, unspecified	N/A^a^
Self-harming issues	Self-harm	Intentional self-harm	N/A	N/A
“Scitzophrenia”	Schizophrenia	Schizophrenia, schizotypal, delusional, and other non-mood psychotic disorder	Schizophrenia	Schizophrenia, unspecified
Schizotypal disorder	Schizotypal disorder	Schizophrenia, schizotypal, delusional, and other non-mood psychotic disorder	Schizotypal disorder	N/A
Mental health issues	Unspecified mental health disorder	Unspecified mental health disorder	N/A	N/A
Postnatal depression	Postpartum depression	Mood (affective) disorders	Major depressive disorder, single episode	Postpartum depression
zoloft	Zoloft	Medications-antidepressants	N/A	N/A
Narcissism	Narcissistic	Disorders of adult personality and behavior	Specific personality disorders	Narcissistic personality disorder
Intermittent explosive disorder	Intermittent explosive disorder	Disorders of adult personality and behavior	Impulse disorders	Intermittent explosive disorder^a^

^a^N/A: not applicable.

^b^“Intermittent explosive disorder” is a fourth level ICD-10 classification; for reporting purposes, we included the fourth level as third level.

Cases in which mental health disorder mentions were more specific, were mapped to lower level *ICD-10* categories. The second and third levels of mapping had 62 and 98 categories, respectively. For example, “paranoid schizophrenia” was classified as “paranoid schizophrenia” at the third level according to the *ICD-10* schema. Since that mention has a third level mapping in the *ICD-10*, this indicated that it can also be mapped backward in the second level (“schizophrenia”) and in the first level (“schizophrenia, schizotypal, delusional, and other non-mood psychotic disorders”). The mapping between levels was done manually by an expert in the field of psychiatry (PS).

A fourth level of *ICD-10* classification (27 categories) was recorded in some narratives. However, for the purpose of reporting the results in our paper, we merged this level with the third classification level. For example, instead of reporting “other impulse disorders” (third level), we included “intermittent explosive disorder” (fourth level) in the third classification level for the representation of results only. Thus, although there were no explicit mentions of “other impulse disorders” (for example), this mapping did not result in any loss of information regarding mentions of mental health disorders. Table 2 shows some examples of extracted mental health disorder mentions mapped into the *ICD-10* schema.

After the mapping of the extracted mental health mentions into the *ICD-10* categories, we eliminated any duplicates at the narrative level. The elimination of duplicates led to narrative level unification since unique mentions of mental health disorders for either victims or POIs were present in each event.

## Results

### Principal Findings

The system was evaluated on a set of 100 unseen, randomly chosen DV events with mentions of mental health disorders. The set was manually inspected and annotated by two domain experts—in DV (CG) and psychiatry (PS)—who identified mentions of mental health disorders for POIs and victims. The interannotator agreement was 90%, calculated as the absolute agreement rate [29], suggesting consistent and reliable annotations by the experts.

Performance of our methodology was evaluated at the narrative level (after the mapping and elimination of any duplicate mental health disorder mentions). We calculated the precision, recall, and F-score for the mental health disorder mentions related to POIs and victims using standard definitions [30] (Multimedia Appendix 3). Table 3 displays the summarized results on the evaluation set, and the performance on the training and development sets.

The F-scores were greater than 80% suggesting reliable results with 87% for mentions related to POIs and 81% for mentions related to victims. Precision ranged from 87% to 97% indicating only a small drop in performance from our development set (1.2%-1.8%). Recall was relatively stable at 79% for the POI (0.3% drop), although for the victim it had a significant drop of 11%, which was expected because our goal was to capture precise mentions of mental health disorders at the narrative level while avoiding noise. It should be noted that victims had fewer mental health disorder mentions at the narrative level when compared to the POIs (36 vs 154, respectively). The false extraction or the nonidentification of a mental health disorder related to a victim affects more the overall extraction performance of the victims than that one of the POIs. Therefore, the values of precision, recall, and F-score for the victims should be taken with caution.

### Large-Scale Corpus Application

Given the relatively accurate results of the methodology to reliably identify mental health disorders, we applied it to all 492,393 DV events. The results revealed 77,995 (15.83%, 77,995/492,393) DV events that involved a mental health disorder mention for either the POI, victim, or both. More than three-quarters (76.96%, 60,032/77,995) of DV events included identified mental health disorders related to POIs versus 16.47% (12,852/77,995) for victims. A total of 5111 (6.55%) DV events had mental health disorders for both the victim and POI (Table 4).

Standardized mental health disorder mentions were grouped into the respective *ICD-10* categories (including our own customized ones) at three levels: first, second, and third. For example, if an event narrative mentioned “antisocial personality disorder,” it was mapped to three levels (third level: antisocial personality disorder; second: specific personality disorders; first: disorders of adult personality and behavior).

**Table 3:** Performance (%) of the system on the evaluation set, the training set, and the development set (100 events each) for the identification of mental health disorder mentions related to victims and persons of interest (POIs) with true positives (TP), false positives (FP), and false negatives (FN).

Set		Precision	Recall	F-score	TP	FP	FN
**Evaluation set**							
	POI	97.5	78.5	86.9	121	3	33
	Victim	87.1	79.0	80.6	27	4	9
**Training set**							
	POI	99.3	84.6	91.3	149	1	27
	Victim	96.1	92.5	94.2	50	2	4
**Development set**							
	POI	98.7	78.8	87.6	164	2	44
	Victim	88.9	90.2	89.5	37	5	4

**Table 4:** Numbers of domestic violence events with identified mentions of mental health disorders for persons of interest (POIs) and victims, and numbers of the mental health disorders for POIs and victims from the large-scale corpus at various levels of the International Classification of Diseases, Tenth Revision (ICD-10).

POI or victim	Events, n	Mental health disorder mentions, n		
		Third level	Second level	First level
POIs only	60,032	21,127	47,831	81,942
Victims only	12,852	7268	14,695	21,290
POIs and victims	5111	N/A^a^	N/A	N/A
Total	77,995	32,479	62,526	103,232

^a^N/A: not applicable.

All mental health disorders were mapped to the first level but not all contained sufficient detail to enable them to be allocated to the second and to the third levels (eg, “unspecified mental disorder,” “intellectual disability, unspecified”). The total number of classified mental health disorder mentions at the first level was 103,232, whereas 62,526 mental health disorder mentions contained sufficient information allowing them to be mapped to the second level, with one-third of mentions (32,479, 31.46%) mapped to the third level (Table 4).

At the first level (Table 5), almost one-third of the 81,942 mentions of mental health disorders (32.46%, 26,598) for the POI and one-fifth (22.79%, 4851) for victims had “unspecified mental health disorders” not explicitly recorded in the narratives by the attending police officer(s). “Mood (affective) disorders” (eg, bipolar disorder, depression) had the highest number of mentions among POIs (15,330, 18.71%) and victims (4946, 23.23%) with “mental and behavioral disorders due to psychoactive substance use” (including alcohol abuse) ranking fourth and fifth for both POIs (6790, 8.29%) and victims (1259, 5.91%), respectively. In all, 12.02% of POIs (9848) and 10.45% of victims (2224) had mentions of “behavioral and emotional disorders with their onset usually occurring in childhood and adolescence” (eg, “attention deficit hyperactivity disorders,” “conduct disorders”) being the third and fourth biggest group of disorders in both POIs and victims. Although mentions of “intellectual disabilities” among POIs (1517, 1.85%) were higher in number than in the victims (939, 4.41%), the rates were higher among victims than POIs. Mentions of traumatic brain injury (eg, “the victim has suffered a brain injury due to a car accident”) were reported for 0.84% of POIs and 1.17% victims (688 and 250 mentions, respectively).

In the second level categories (Table 6), “alcohol abuse” was the second highest mental health disorder among POIs (5829, 12.19%) and the fifth highest reported among victims (1180, 8.03%) reinforcing the established link between DV and alcohol use [31-33]. Additionally, there were 644 victims with “dementia, unspecified” (4.38%, 644/14,609) and 546 POI ones (1.14%, 546/47,600).

**Table 5:** Number of events containing mental health disorders grouped according to the first level of mental health disorder categories (from the International Classification of Diseases, Tenth Revision [ICD-10]) for both persons of interest (POIs) and victims from 492,393 domestic violence events as recorded by the New South Wales Police Force in Australia between the 2005 and 2016 period.

Mental health disorders (first level)	Mentions, n	
	POI	Victim
Unspecified mental disorder	26,598	4851
Mood (affective) disorders	15,330	4946
Behavioral and emotional disorders with onset usually occurring in childhood and adolescence	9848	2224
Anxiety, dissociative, stress-related, somatoform, and other nonpsychotic mental disorders	3755	2261
Mental and behavioral disorders due to psychoactive substance use	6790	1259
Schizophrenia, schizotypal, delusional, and other non-mood psychotic disorders	5771	1032
Intentional self-harm	3271	949
Intellectual disability	1517	939
Mental disorders due to known physiological conditions	559	649
Pervasive and specific developmental disorders	1775	485
Disorders of adult personality and behavior	1340	420
Substance abuse	2852	370
Injury of unspecified body region	800	265
Traumatic brain injury	688	250
Medications-antidepressants	400	130
Symptoms, signs, and abnormal clinical and laboratory findings	189	79
Other degenerate diseases of the nervous system	62	52
Chromosomal abnormalities, not elsewhere classified	53	39
Medications-anxiety	91	24
Behavioral syndromes associated with physiological disturbances and physical factors	31	23
Medications-antipsychotics	142	16
Unspecified drug-induced disorders	57	1
Drug prescription abuse	5	1
Medications-neuroleptics	1	0
Systematic atrophies primarily affecting the central nervous system	11	6
Unspecified diseases of the nervous system	6	3

**Table 6:** The 20 most common mental health disorder mentions (at the second level of the International Classification of Diseases, Tenth Revision [ICD-10]) for both persons of interest (POIs) and victims from 492,393 domestic violence events as recorded by the New South Wales Police Force in Australia between the 2005 and 2016 period.

Mental health disorders (second level)	Mentions, n	
	POIs	Victims
Major depressive disorder, single episode	8944	3269
Alcohol abuse	5829	1180
Bipolar disorder	5449	1553
Other behavioral and emotional disorders with onset usually occurring in childhood and adolescence	4888	776
Schizophrenia	4852	849
Attention deficit hyperactivity disorder	3980	1312
Other anxiety disorders	2446	1714
Pervasive developmental disorder	1721	477
Specific personality disorders	1310	372
Intellectual disability, unspecified	1225	779
Conduct disorders	903	121
Injury of unspecified body region	800	265
Reaction to severe stress, and adjustment disorders	790	388
Persistent mood disorder	781	90
Unspecified psychosis not due to a substance or known physiological condition	648	124
Dementia, unspecified	546	644
Other psychoactive substance related disorders^a^	370	23
Obsessive-compulsive disorder	314	81
Other stimulant related disorders^a^	248	18
Cannabis abuse^a^	234	18
Intellectual disability, mild^b^	153	83
Symptoms and signs involving emotional state^b^	189	79
Intellectual disability, severe^b^	61	51

^a^Mental health disorders that are not in the top 20 for victims.

^b^Mental health disorders that are not in the top 20 for POIs.

In the third level categories (Table 7), “bipolar disorder, unspecified” ranked first in mentions for both POIs (5445, 21.59%) and victims (1553, 21.36%) with similar rates. However, it was observed that in POIs that “unspecified behavioral and emotional disorders with onset usually occurring in childhood and adolescence” were second in mentions (4888, 19.38%) unlike with victims that had “anxiety disorder, unspecified” (1459, 20.07%).

**Table 7:** The 20 most common mental health disorder mentions (at the third level of the International Classification of Diseases, Tenth Revision [ICD-10] categories) in 492,393 domestic violence events as recorded by the New South Wales Police Force in Australia between the 2005 and 2016 period.

Mental health disorders (third level)	Mentions, n	
	POIs	Victims
Bipolar disorder, unspecified	5445	1553
Unspecified behavioral and emotional Disorders with onset usually occurring in childhood and adolescence	4888	776
Schizophrenia, unspecified	4630	821
Anxiety disorder, unspecified	2336	1459
Autism	956	329
Oppositional defiant disorder	811	114
Suicide attempt	800	265
Cyclothymic disorder	780	90
Posttraumatic stress disorder	767	379
Asperger syndrome	758	146
Paranoid personality disorder	638	157
Obsessive-compulsive disorder, unspecified	314	81
Personality disorder, unspecified	299	102
Borderline personality disorder	271	92
Postpartum depression	261	265
Paranoid schizophrenia^a^	249	28
Suicidal ideations	189	79
Dissociative identity disorder	143	44
Panic disorder	104	253
Conduct disorder, unspecified^a^	92	7
Alzheimer’s disease, unspecified^b^	54	51
Down syndrome, unspecified^b^	53	39

^a^Mental health disorders that are not in the top 20 for victims.

^b^Mental health disorders that are not in the top 20 for POIs.

## Discussion

### Overview

Text mining the police event narratives yielded a rich vein of data on the mental health status of victims and POIs involved in DV events that could be useful in policy formulation and prevention that to date has been unavailable. By mining a large cohort of DV police events, we identified many mental health disorder mentions for both the POIs and the victims highlighting the possible role of mental health disorders in DV. Studies have shown that mental illness can increase the likelihood of being in an abusive relationship [3,9], which is consistent with the higher prevalence of mental health disorder mentions among victims (16%).

We aimed to recognize and assign mental health disorders to the POIs and the victims involved in a DV event at the narrative level. Therefore, our rules were focused on precision in order to enable the assignment of the respective disorders to either the POIs or the victims. Many mental health mentions in a single narrative were (varied) mentions of the same disorder for the same individual. This explains the high precision (87.1%-97.5%) when compared to recall (78.5%-79.0%).

### Error Analysis

We inspected the evaluation set for sources of false positive and false negative errors in the extraction of mental health disorder mentions. There was a limited number of false positives for either the POI’s or victim’s mental health disorder mentions. In some cases, the lexical patterns used in the rules were ambiguous and assigned a mental health disorder to the wrong person. For example, in the following sentence, “POI has the potential to become violent with the victims due to her alcoholism,” “alcoholism” was extracted incorrectly as a mental health disorder for the victim instead of the POI. In other instances, the specific mention did not refer to an actual mental health problem and the rules incorrectly identified a mental health disorder mention due to the ambiguous nature of a specific situation that mapped to a term in the mental health disorder dictionary (eg, “As a result of the glass on the floor the defendant had cut herself [false positive for POI]”).

In one-third of the false negative cases (33%), the lexical patterns had not been incorporated as they were previously unseen in the training and development sets (eg, “There has been a history of alcohol abuse [false negative: mental health disorder mention for POI] and malicious damage perpetrated by the accused,” “The victim also stated to police that during her time with the POI she was intoxicated as she has an alcohol addiction [false negative: mental health disorder mention for victim]”). Additionally, in almost 40% of false negatives, the rules ignored the correct mental health disorder mention related to either the POI or the victim due to the lack of a semantic anchor specifying the role of the individual (eg, “XXX [name of victim] was admitted to YYY house for depression and anorexia [false negative: mental health disorder mention for victim],” “Her child’s behavior is because of a condition ADHD [false negative: mental health disorder mention for POI]”). In such cases, we chose not to engineer any rules in order to protect the system’s precision and avoid the generation of false positives for potentially other individuals (eg, witnesses, children at risk, friend, neighbor) that could be involved in a DV event and suffering from a mental disorder.

### Limitations and Future Work

We designed the rules after inspecting and exploring a relatively small training and development set. However, these sets contained significant numbers of mental health disorder mentions (Table 3). Still, the total number of victim mentions in the evaluation set was significantly lower (almost three times lower) than for POI, which may explain the relatively lower performance for the victim mentions. It is possible that a set focusing only on victim mentions (as opposed to a set that has mental health mentions for either POIs or victims) might have helped to cast a wider net of rules for the identification of the mental health disorders for the victims. Since we based our rules on common lexical patterns, they potentially could be used to process similar types of police-recorded narratives (eg, sexual assaults and other recorded crimes). Although the rules might work on other data, they could require further adjustments both in lexical and dictionary coverage (eg, identification of non-mental health diseases).

We were unaware if the extracted mentions of mental health disorders are valid as they were recorded by police officers who are not expert in mental health and therefore caution is warranted when interpreting the findings. Information on mental health status can be provided to the police by victims, POIs, and witnesses. We plan to examine the veracity of these “informal” mentions of mental health disorders by using formal diagnoses contained in administrative data collections.

We will also expand our set of targeted information from the police narratives in order to assess the characteristics of the POIs and victims for risk groups such as the elderly, those in same sex relationships, and those in carer relationships. The extracted information can be used in designing predictive models to investigate whether we can predict DV recurrent events for groups at risk and inform prevention strategies.

### Conclusions

We have designed, implemented, and evaluated a rule-based approach for the extraction of mental health disorders for both POIs and victims involved in DV events as recorded by the NSWPF in event narratives that could not be examined manually on a large scale. Performance was promising, with precision of 87.1% for the victims and 97.5% for the POIs. The results are encouraging and indicate that automated text-mining methods can be used to extract important information from police narratives with reasonable performance. The information extracted from a large-scale set of DV reports allowed us to identify and confirm patterns and links between DV events and mental health disorders. The identified information can be used for further research that aims to assess the characteristics and features of victims and POIs involved in DV events.

## Appendix

Multimedia Appendix 1 A hypothetical example of a domestic violence event narrative as recorded by the New South Wales Police Force.

Multimedia Appendix 2 The International Classification of Diseases, Tenth Revision (ICD-10) Mental and Behavioural Disorders schema used to map the extracted and standardised mental health disorder mentions containing three levels (first, second and third).

Multimedia Appendix 3 Evaluation metrics used for our method. True positive (TP) is the detection of a correct mention of a mental health disorder in an event. False positive (FP) is the extraction of any unrelated mention that has not been annotated manually. False negative (FN) is the correct mental health disorder mention that was not detected by the method. True negative (TN) is when the method did not identify any mental health disorder mentions where none has been annotated. Performance of the system was calculated using the standard definitions of precision (the number of TP against the number of TP and FN), recall (the number of TP against the number of FN and TP), and F-score (the harmonic mean between precision and recall [31]).

## Abbreviations

COPS: Computerised Operational Policing System
DV: domestic violence
GATE: General Architecture for Engineering
ICD-10: International Classification of Diseases, Tenth Revision
JAPE: Java Annotations Pattern Engine
NSMHWB: National Survey of Mental Health and Wellbeing
NSWPF: New South Wales Police Force
WHO: World Health Organization
