# Correction: Automatic Extraction of Mental Health Disorders From Domestic Violence Police Narratives: Text Mining Study

**DOI:** 10.2196/13007

**Published:** 2019-04-05

**Authors:** George Karystianis, Armita Adily, Peter Schofield, Lee Knight, Clara Galdon, David Greenberg, Louisa Jorm, Goran Nenadic, Tony Butler

**Affiliations:** 1 Kirby Institute Faculty of Medicine University of New South Wales Sydney Australia; 2 Neuropsychiatry Service Hunter New England Health Newcastle Australia; 3 Victims Services New South Wales Department of Justice Sydney Australia; 4 School of Psychiatry University of New South Wales Sydney Australia; 5 Centre for Big Data Research in Health University of New South Wales Sydney Australia; 6 School of Computer Science University of Manchester Manchester United Kingdom

The authors of “Automatic Extraction of Mental Health Disorders From Domestic Violence Police Narratives: Text Mining Study” (J Med Internet Res 2018;20(9):e11548) incorrectly under-reported the number of ICD-10 level 1, 2 and 3 categories used for mapping the identified mental health disorder mentions and the number of domestic violence events at certain mental health disorders in the Abstract and in the Results section. Note that the acronym “POI(s)” is frequently used within the text to mean “person(s) of interest”; this use has been preserved in quoted sections of text below.

In the Abstract, the authors have incorrectly listed the number of domestic violence events with depression in victims as “22.30%; 3258” instead of “22.25%, 3269” and in POIs as “18.73%, 8918” instead of “18.70%, 8944”. Additionally, the alcohol abuse domestic violence event percentage for POIs were incorrectly listed as “12.24%, 5829” instead of “12.19%, 5829” and the percentage and number of domestic violence events for various anxiety disorders for victims were incorrectly listed as “11.43%, 1671” instead of “11.66%, 1714”. All aforementioned errors have been corrected.

In the “Data” subsection of the Methods, the authors have listed a total of 24 categories instead of 26 in Textbox 1. The authors with to add two more categories that were omitted: “25. Unspecified diseases of the nervous system: eg, neurological disorder” and “26. Systemic atrophies primarily affecting the central nervous system: eg, Huntington disease”. The authors wish to remove the example “Huntington disease” from the 15 category level 1 “Diseases of the nervous system: eg, Alzheimer disease, Huntington disease, frontotemporal dementia”. Also, the authors did not write down correctly the name of the category 15 and they wish to change its name from “Diseases of the nervous system” to “Other degenerate diseases of the nervous system”.

In the “Mapping of Extracted Mental Health Disorder Mentions to the International Classification of Diseases, Tenth Revision” subsection of the Methods, the authors incorrectly stated that “The first level was the most generic (24 categories)” instead of “26 categories”; “The second and third levels of mapping had 71 and 163 categories, respectively” instead of “62 and 98 categories”; and “a fourth level of *ICD-10* classification (25 categories) was recorded” instead of “a fourth level of *ICD-10* classification (27 categories)”. All aforementioned errors have been corrected.

The authors have incorrectly reported the number of certain mental health disorder mentions at the first, second and third levels in the “Large-Scale Corpus Application” subsection of the Results. In Table 4, the authors listed the number of mental health disorder mentions in the “POIs only” row as “25,211” instead of “21,127” at the third level; “47,600” instead of “47,831” at the second level; and “79,727” instead of “81,942” at the first level. The authors incorrectly reported that the number of mental health disorder mentions for the “Victims only” as “14,609” instead of “14,695” at the second level and as “20,774” instead of “21,290” at the first level. The authors incorrectly reported the number of mental health disorder mentions in the “Total” row as “62,209” instead of “62,526“ at the second level and “100,501” instead of “103,232” at the first level. All aforementioned errors have been corrected.

Subsequently, the same errors were listed in the third paragraph of the “Large-Scale Corpus Application” subsection which mentions Table 4, and the number of classified mental health disorder mentions have changed to reflect this:

The total number of classified mental health disorder mentions at the first level was 103,232, whereas 62,526 mental health disorder mentions contained sufficient information allowing them to be mapped to the second level, with one-third of mentions (32,479, 31.46%) mapped to the third level (Table 4).

The number of mentions for persons of interest and victims in Tables 5 and 6 for certain mental health disorders have been listed incorrectly by the authors.

In Table 5, “Diseases of the nervous system” has been changed to “Other degenerate diseases of the nervous system”. The authors wish to change the number of mentions in the “POIs” column for the following mental health disorders:

- The value for “Mood (affective) disorders” has been changed from “14,566” to “15,330”
- The value for “Behavioral and emotional disorders with onset usually occurring in childhood and adolescence” has been changed from “9034” to “9848”
- The value for “Anxiety, dissociative, stress-related, somatoform, and other nonpsychotic mental disorders” has been changed from “3518” to “3755”
- The value for “Mental and behavioral disorders due to psychoactive substance use” has been changed from “6762” to “6790”
- The value for “Schizophrenia, schizotypal, delusional, and other non-mood psychotic disorders” has been changed from “5598” to “5771”
- The value for “Intellectual disability” has been changed from “1444” to “1517”
- The value for “Mental disorders due to known physiological conditions” has been changed from “557” to “559”
- The value for “Pervasive and specific developmental disorders” has been changed from “1686” to “1775”
- The value for “Disorders of adult personality and behavior” has been changed from “1308” to “1340”
- The value for “Other degenerate diseases of the nervous system” has been changed from “78” to “62”
- The value for “Behavioral syndromes associated with physiological disturbances and physical factors” has been changed from “29” to “31”

Also in Table 5, the authors wish to change the number of mentions in the “Victims” column for the following mental health disorders:

- The value for “Mood (affective) disorders” has been changed from “4734” to “4946”
- The value for “Behavioral and emotional disorders with onset usually occurring in childhood and adolescence” has been changed from “2143” to “2224”
- The value for “Anxiety, dissociative, stress-related, somatoform and other nonpsychotic mental disorders” has been changed from “2113” to “2261”
- The value for “Schizophrenia, schizotypal, delusional, and other non-mood psychotic disorders” has been changed from “1020” to “1032”
- The value for “Intellectual disability” has been changed from “907” to “939”
- The value for “Mental disorders due to known physiological conditions” has been changed from from “648” to “649”
- The value for “Disorders of adult personality and behavior” has been changed from “406” to “420”
- The value for “Other degenerate diseases of the nervous system” has been changed from “61” to “52”

The authors wish to add two new rows to Table 5:

- “Systematic atrophies primarily affecting the central nervous system” (POIs=11; victims=6)
- “Unspecified diseases of the nervous system” (POIs=6; victims=3)

Consequently, the same errors were listed in the fourth paragraph of the “Large-Scale Corpus Application” subsection which mentions Table 5, and the number of classified mental health disorder mentions have been changed to reflect this:

At the first level (Table 5), almost one-third of the 81,942 mentions of mental health disorders (32.46%, 26,598) for the POI and one-fifth (22.79%, 4851) for victims had “unspecified mental health disorders” not explicitly recorded in the narratives by the attending police officer(s). “Mood (affective) disorders” (eg, bipolar disorder, depression) had the highest number of mentions among POIs (15,330, 18.71%) and victims (4946, 23.23%) with “mental and behavioral disorders due to psychoactive substance use” (including alcohol abuse) ranking fourth and fifth for both POIs (6790, 8.29%) and victims (1259, 5.91%), respectively. In all, 12.02% of POIs (9848) and 10.45% of victims (2224) had mentions of “behavioral and emotional disorders with their onset usually occurring in childhood and adolescence” (eg, “attention deficit hyperactivity disorders,” “conduct disorders”) being the third and fourth biggest group of disorders in both POIs and victims. Although mentions of “intellectual disabilities” among POIs (1517, 1.85%) were higher in number than in the victims (939, 4.41%), the rates were higher among victims than POIs. Mentions of traumatic brain injury (eg, “the victim has suffered a brain injury due to a car accident”) were reported for 0.84% of POIs and 1.17% victims (688 and 250 mentions, respectively).

In Table 6, the authors wish to change the number of mentions in the “POIs” column for the following mental health disorders:

- The value for “Major depressive disorder, single episode” has been changed from “8918” to “8944”
- The value for “Bipolar disorder” has been changed from “5448” to “5449”
- The value for “Other behavioral and emotional disorders with onset usually occurring in childhood and adolescence” has been changed from “4888” to “4880”
- The value for “Other anxiety disorders” has been changed from “2420” to “2446”
- The value for ”Pervasive developmental disorder” has been changed from “1635” to “1721”
- The value for “Specific personality disorders” has been changed from “1279” to “1310”
- The value for “Conduct disorders” has been changed from “877” to “903”
- The value for “Reaction to severe stress, and adjustment disorders” from “787” to “790”.

Also in Table 6, the authors wish to replace the number of mentions in the “Victims” column for the following mental health disorders:

- The value for “Major depressive disorder, single episode” has been changed from “3258” to “3269”
- The value for “Schizophrenia” has been changed from “846” to “849”
- The value for “Other anxiety disorders” has been changed from “1671” to “1714”
- The value for “Pervasive developmental disorder” has been changed from “462” to “477”
- The value for “Specific personality disorders” has been changed from “358” to “372”
- The value for “Conduct disorders” has been changed from “119” to “121”

Consequently, the same errors were listed in the fifth paragraph of the “Large-Scale Corpus Application” subsection which mentions Table 6, and the number of mentions for mental health disorders for both persons of interest and victims have been changed to reflect this:

In the second level categories (Table 6), “alcohol abuse” was the second highest mental health disorder among POIs (5829, 12.19%) and the fifth highest reported among victims (1180, 8.03%) reinforcing the established link between DV and alcohol use [31-33]. Additionally, there were 644 victims with “dementia, unspecified” (4.38%, 644/14,609) and 546 POI ones (1.14%, 546/47,600).

The authors wish to state that the requested changes do not affect the reported conclusions on the paper. Since the number of mental health disorder mentions in domestic violence events was slightly under-reported, the conclusions remain the same. The only difference is the increase of certain mentions of mental health disorders. The original statistics that were reported were marginally different in the last out of two decimals.

The correction will appear in the online version of the paper on the JMIR website on April 5, 2019, together with the publication of this correction notice. Because this was made after submission to PubMed, PubMed Central, and other full-text repositories, the corrected article also has been resubmitted to those repositories.

