# Supplementary figures and images for: Automatic Extraction of Mental Health Disorders From Domestic Violence Police Narratives: Text Mining Study
Source: J Med Internet Res. 2018 Sep 13;20(9):e11548. doi: 10.2196/11548 (PMC6231811; doi:10.2196/11548)

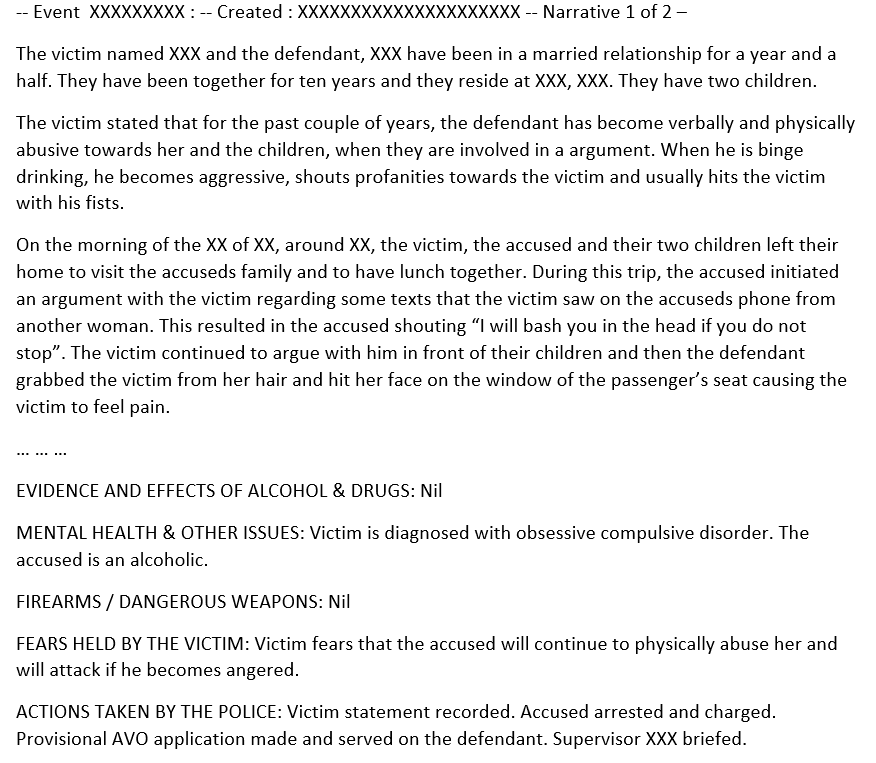

Supplement: Multimedia Appendix 1 [file jmir_v20i9e11548_app1.png]

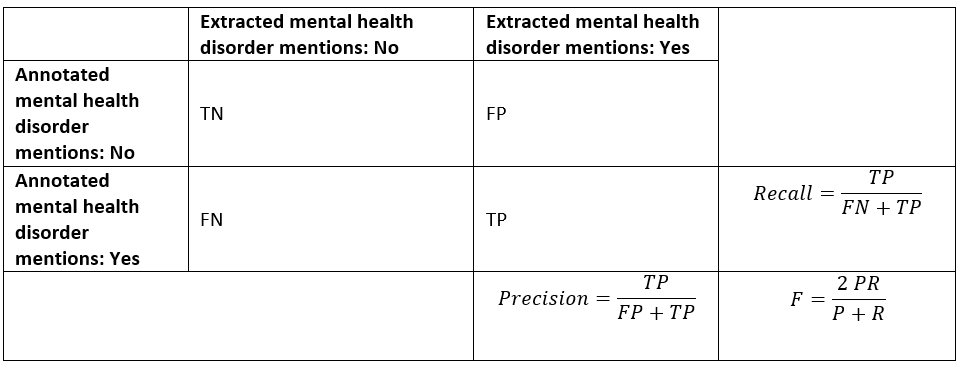

Supplement: Multimedia Appendix 3 [file jmir_v20i9e11548_app3.png]
