# Supplementary material for: Automatic Extraction of Mental Health Disorders From Domestic Violence Police Narratives: Text Mining Study
Source: J Med Internet Res. 2018 Sep 13;20(9):e11548. doi: 10.2196/11548 (PMC6231811; doi:10.2196/11548)
Supplement: Multimedia Appendix 2 [file jmir_v20i9e11548_app2.pdf]

Multimedia Appendix 1

Table 1: The ICD-10 Mental and Behavioural Disorders schema used to map the extracted and standardised mental health disorder mentions containing three levels (first, second and third).

First level	Second level	Third level	Fourth level
Mental disorders due to known physiological conditions	Vascular dementia	-	-
	Unspecified dementia	-	-
	Delirium	-	-
	Unspecified mental disorder due to known physiological condition	-	-
Mental and behavioral disorders due to psychoactive substance use	Alcohol related disorders	-	-
	Opioid related disorders	-	-
	Cannabis related disorders	-	-
	Cocaine related disorders	-	-
	Other stimulant related disorders	-	-
	Nicotine dependence	-	-
	Other psychoactive substance related disorders	-	-
Schizophrenia, schizotypal, delusional, and other non-mood psychotic disorders	Schizophrenia	Paranoid schizophrenia	-
		Disorganized schizophrenia	-
		Catatonic schizophrenia	-
		Undifferentiated schizophrenia	-
		Residual schizophrenia	-
		Other schizophrenia	-
		Unspecified schizophrenia	-
	Schizotypal disorder	-	-
	Delusional disorders	-	-
	Brief psychotic disorder	-	-
	Shared psychotic disorder	-	-
	Schizoaffective	-	-
	Unspecified psychosis not due to a substance or known physiological condition	-	-
Mood [affective] disorders	Manic episode	-	-
	Bipolar disorder	Bipolar disorder, unspecified	-
		Other bipolar disorders	Bipolar II disorders
	Major depressive disorder,	Postpartum depression	-

	single episode		
	Major depressive disorder, recurrent	Other recurrent depressive disorders	
	Persistent mood disorders	Cyclothymic disorder	-
		Dysthymic disorder	-
		Other persistent mood disorders	Disruptive mood dysregulation disorder
	Unspecified mood disorder	-	-
Anxiety, dissociative, stress-related, somatoform and other nonpsychotic mental disorders	Phobic anxiety disorder	Agoraphobia	-
		Social phobias	Social phobia, generalised
		Specific isolated phobias	Arachnophobia
			Claustrophobia
			Acrophobia
			Androphobia
			Gynaecophobia
		Other phobic anxiety disorders	-
		Phobic anxiety disorder, unspecified	-
	Other anxiety disorders	Panic disorder	-
		Generalised anxiety disorder	-
		Anxiety disorder, unspecified	-
	Obsessive compulsive disorders	Hoarding disorder	-
		Excoriation disorder	-
		Obsessive compulsive disorder, unspecified	-
	Reaction to severe stress and adjustment disorders	Acute stress reaction	-
		Post-traumatic stress disorder	-
		Adjustment disorders	-
	Dissociative and conversion disorders	Dissociative amnesia	-
		Dissociative fugue	-
		Dissociative stupor	-
		Other dissociative and conversion disorders	Dissociative identity disorder
		Dissociative and conversion disorder, unspecified	-
	Somatoform disorders	Somatization disorder	-
		Undifferentiated somatoform disorder	-

		Hypochondrial disorders	Body dysmorphic disorder
			Hyponhondriasis
	Other nonpsychotic mental disorders	Depersonalization-derealization syndrome	-
		Pseudobulbar affect	-
		Nonpsychotic mental disorder, unspecified	-
Behavioral syndromes associated with physiological disturbances and physical factors	Eating disorders	Anorexia nervosa	-
		Bulimia nervosa	-
		Other eating disorders	Binge eating disorder
			Avoidant food intake disorder
	Sleep disorders not due to a substance or known physiological condition	Insomnia not due to a substance or known physiological condition	Primary insomnia
			Adjustment insomnia
			Paradoxical insomnia
			Psychophysiologic insomnia
	Sexual dysfunction not due to a substance or known physiological condition	Hypoactive sexual desire disorder	-
	Abuse of non-psychoactive substances	Abuse of steroids or hormones	-
Disorders of adult personality and behavior	Specific personality disorders	Paranoid personality disorder	-
		Schizoid personality disorder	-
		Antisocial personality disorder	-
		Borderline personality disorder	-
		Histrionic personality disorder	-
		Obsessive compulsive personality disorder	-
		Avoidant personality disorder	-
		Dependent personality disorder	-
		Other specific personality disorders	Narcissistic personality disorder
		Personality disorder, unspecified	-
	Impulse disorders	Pathological gambling	-
		Pyromania	-
		Kleptomania	-
		Trichotillomania	-
		Other impulse disorders	Intermittent explosive disorder
		Impulse disorder, unspecified	-

	Gender identity disorders	Transsexualism	-
		Dual role transsexualism	-
		Gender identify disorder	-
	Paraphilias	Fetishism	-
		Transvestic fetishism	-
		Exhibitionism	-
		Voyeurism	-
		Paedophilia	-
		Sadomasochism	-
		Other paraphilias	Frotteurism
	Other personalities of adult and personality behaviour	Factitious disorder	-
	Unspecified disorder of adult personality and behavior	-	-
Intellectual disabilities	Mild intellectual disabilities	-	-
	Moderate intellectual disabilities	-	-
	Severe intellectual disabilities	-	-
	Profound intellectual disabilities	-	-
	Unspecified intellectual disabilities	-	-
Pervasive and specific developmental disorders	Specific developmental disorders of speech and language	Phonological disorder	-
		Expressive language disorder	-
		Mixed receptive-expressive language disorder	-
		Other developmental disorders of speech and language	Childhood onset fluency disorder
			Social pragmatic communication disorder
	Specific developmental disorders of scholastic skills	Specific reading disorder	-
		Mathematics disorder	-
		Other developmental disorders of scholastic skills	Disorder of written expression
	Pervasive developmental disorders	Autism	-
		Rett’s syndrome	-
		Asperger’s syndrome	-
		Pervasive developmental disorder, unspecified	-
	Unspecified disorder of psychological development	-	-

Behavioral and emotional disorders with onset usually occurring in childhood and adolescence	Attention-deficit hyperactivity disorders	-	-
	Conduct disorders	Conduct disorder, unspecified	-
		Oppositional defiant disorder	-
	Emotional disorders with onset specific to childhood	Separation anxiety disorder of childhood	-
	Disorders of social functioning with onset specific to childhood and adolescence	Selective mutism	-
		Reactive attachment disorder of childhood	-
		Disinhibited attachment disorder of childhood	-
	Tic disorder	Transient tic disorder	-
		Chronic motor or vocal tic disorder	-
		Tourette's disorder	-
	Other behavioural and emotional disorders	Unspecified behavioural and emotional disorders	-
Unspecified mental disorder			-
Other degenerative diseases of the nervous system	Alzheimer’s disease	Alzheimer’s disease, unspecified	-
	Other degenerative diseases of the nervous system, not elsewhere classified	Frontotemporal dementia	-
Systemic atrophies primarily affecting the central nervous system	Huntington’s disease	-	-
Injury of unspecified body region	Injury of unspecified body region	Unspecified injury	Suicide attempt
Symptoms and signs involving cognition, perception, emotional state and behavior	Symptoms and signs involving emotional state	Other symptoms and signs involving emotional state	Homicidal and suicidal ideations
Chromosomal abnormalities, not elsewhere classified	Down syndrome	Down syndrome, unspecified	-
Intentional self-harm	-	-	-
Unspecified diseases of the nervous system	-	-	-
unspecified drug induced disorders	-	-	-
Medications - neuroleptics	-	-	-
Medications - antipsychotics	-	-	-
Medications – anti anxiety	-	-	-
Medications – antidepressants	-	-	-
Traumatic brain injury	-	-	-

Substance abuse	-	-	-
Drug prescription abuse	-	-	-
